# Identification of Critical Genes for Recurrent Aphthous Ulcer by Transcriptome Data Analysis and Mendelian Randomization

**DOI:** 10.3290/j.ohpd.c_2799

**Published:** 2026-09-09

**Authors:** Xingguo Fu, Haishan Zhang, Yaomei Li, Andi Li, Qiulin Liu, Xiaojuan Zeng

**Affiliations:** a Xingguo Fu* Medical Student, College & Hospital of Stomatology, Guangxi Medical University, Nanning, Guangxi, China; Guangxi Health Commission Key Laboratory of Prevention and Treatment for Oral Infectious Diseases, College & Hospital of Stomatology, Guangxi Medical University, Nanning, Guangxi, China; Guangxi Key Laboratory of Oral and Maxillofacial Rehabilitation and Reconstruction, College & Hospital of Stomatology, Guangxi Medical University, Nanning, Guangxi, China. Led the research design and developed the overall framework, conducted the literature review, formulated research hypotheses, and wrote the initial draft.; b Haishan Zhang* PhD Student, College & Hospital of Stomatology, Guangxi Medical University, Nanning, Guangxi, China; Guangxi Health Commission Key Laboratory of Prevention and Treatment for Oral Infectious Diseases, College & Hospital of Stomatology, Guangxi Medical University, Nanning, Guangxi, China; Guangxi Key Laboratory of Oral and Maxillofacial Rehabilitation and Reconstruction,College & Hospital of Stomatology, Guangxi Medical University, Nanning, Guangxi, China. Managed data collection and performed statistical analysis.; c Yaomei Li Medical Student, College & Hospital of Stomatology, Guangxi Medical University, Nanning, Guangxi, China; Guangxi Health Commission Key Laboratory of Prevention and Treatment for Oral Infectious Diseases, College & Hospital of Stomatology, Guangxi Medical University, Nanning, Guangxi, China; Guangxi Key Laboratory of Oral and Maxillofacial Rehabilitation and Reconstruction, College & Hospital of Stomatology, Guangxi Medical University, Nanning, Guangxi, China. Assisted with the literature review and provided analytical support.; d Andi Li PhD Student, College & Hospital of Stomatology, Guangxi Medical University, Nanning, Guangxi, China; Guangxi Health Commission Key Laboratory of Prevention and Treatment for Oral Infectious Diseases, College & Hospital of Stomatology, Guangxi Medical University, Nanning, Guangxi, China; Guangxi Key Laboratory of Oral and Maxillofacial Rehabilitation and Reconstruction, College & Hospital of Stomatology, Guangxi Medical University, Nanning, Guangxi, China. Contributed to writing and revising the manuscript for clarity.; e Qiulin Liu Dentist, College & Hospital of Stomatology, Guangxi Medical University, Nanning, Guangxi, China. Offered methodological guidance and helped interpret the results.; f Xiaojuan Zeng Professor, College & Hospital of Stomatology, Guangxi Medical University, Nanning, Guangxi, China. Provided feedback on the manuscript and ensured final proofreading. *These authors contributed equally to this work.

**Keywords:** differentially expressed genes, immune regulation, recurrent aphthous ulcer, Mendelian randomization, transcriptomics.

## Abstract

**Purpose:**

Recurrent aphthous ulcer (RAU) is a common oral mucosal disorder with a poorly understood etiology, significantly affecting patients’ quality of life. This study aims to investigate critical genes linked to RAU and explore their biological mechanisms using transcriptomic data and Mendelian randomization (MR) analysis.

**Materials and Methods:**

RAU-related gene expression data from the GEO database (GSE37265) were analyzed to identify differentially expressed genes (DEGs). A two-sample MR approach was used to assess the causal impact of expression quantitative trait loci (eQTL) on RAU. Critical genes were identified by intersecting DEGs with significant MR findings. GO and KEGG pathway enrichment analyses were performed, along with GSEA and immune cell infiltration analysis, to investigate the functions and mechanisms of these genes in RAU.

**Results:**

A total of 184 differentially expressed genes (DEGs) were identified, while 339 RAU-associated genes were screened through MR analysis. Cross-validation further identified 7 critical genes. Among these, CCR1, ERP27, HCK, MICB, and SLC2A3 showed protective associations with RAU risk, whereas CD177 and IFITM1 were positively associated with increased risk. Enrichment analysis revealed that these genes are involved in specific biological processes, including cell migration, immune response, and metabolic regulation, which are closely linked to RAU pathogenesis.

**Conclusion:**

This systematic study comprehensively investigates the critical causative genes underlying RAU, emphasizing the intricate relationships between immune regulation and metabolic disturbances in its pathology. These findings lay a solid foundation for the development of novel biomarkers and may inform future research on targeted therapeutic strategies for RAU.

Recurrent aphthous ulcer (RAU), commonly known as recurrent oral ulcer (ROU) or recurrent aphthous stomatitis (RAS), is the most frequent ulcerative disorder of the oral mucosal tissues. Its prevalence is approximately 25%. RAU is more common in women than in men and can occur at any age.^4^ Although RAU is not life-threatening, its recurrent episodes, prolonged healing and severe pain can seriously affect patients’ normal diet and daily life. The precise cause of RAU remains uncertain and is thought to involve multiple contributing factors, such as stress, physical or chemical injuries, infections, allergies, genetic predisposition, and deficiencies in certain nutrients.^29,33^ There is currently no standardized treatment protocol for RAU. Existing approaches mainly focus on symptom relief and recurrence prevention. However, they do not provide a definitive cure.^10,18^ Therefore, an in-depth understanding of the molecular mechanisms of RAU is crucial for the development of new diagnostic markers and therapeutic strategies.

In recent years, bioinformatics analysis has been widely used in the field of medicine, and the development of high-throughput gene chips and transcriptome sequencing technology has provided new directions for revealing the pathophysiological mechanisms of diseases. In the study of genetic factors, the Mendelian Randomization (MR) approach has emerged as a pivotal method. It utilizes single nucleotide polymorphisms (SNPs) as instrumental variables (IVs) and leverages data from genome-wide association studies (GWAS) to investigate causal relationships.^17^

Expression quantitative trait loci (eQTL) studies advance our understanding of how genetic variations impact gene expression and related complex traits.^40^ By correlating specific SNPs with gene expression levels, eQTL analyses can clarify genetic contributions to disease pathology.^26^

This study integrates bioinformatics with MR techniques to pinpoint critical genes associated with RAU. We further investigate the genetic mechanisms underpinning RAU through enrichment and immune cell infiltration analyses. These analyses may help identify novel therapeutic targets and improve our understanding of the molecular basis of the disease.

## MATERIALS AND METHODS

## Dataset Data Source

In this study, the gene microarray dataset GSE37265, associated with RAU, was obtained from the Gene Expression Omnibus (GEO) database (https://www.ncbi.nlm.nih.gov/geo/). This dataset includes 14 normal control samples and 14 RAU lesion tissue samples.

## Identification of Differentially Expressed Genes (DEGs)

Gene expression differences between normal and RAU samples in the GSE37265 dataset were analyzed using the R package limma. A linear model was applied, combined with a Bayesian method, to compute t-values and log-fold changes (logFC). Differentially expressed genes (DEGs) were identified based on the filtering criteria of |logFC| ≥ 2 and an adjusted p<0.05. We selected this relatively stringent threshold to focus on genes with both strong effect sizes and robust statistical significance, thereby reducing false-positive findings and highlighting the most biologically meaningful transcriptional changes. Considering the limited sample size and the exploratory nature of this study, a stricter cutoff was considered more appropriate for downstream analyses. Visualization of the identified DEGs was performed through volcano plots generated with the ggplot2 package and heatmaps created using the pheatmap package.

## eQTL Analysis of Exposure Data

eQTL analysis is a method for studying the association between SNPs and gene expression by considering gene expression as a trait. In this research, eQTL-related GWAS summary statistics were downloaded from the IEU Open GWAS project, and a total of 19,942 eQTL datasets were included as exposure variables for the study (https://gwas.mrcieu.ac.uk/). Strong instrumental SNPs were selected using the R package Two-Sample MR. (1) SNPs statistically significantly associated with eQTL were screened by a p-value threshold (P1 < 5e-8). (2) The linkage disequilibrium parameter was set to r^2^ < 0.001, and the aggregation distance was set to 10 ,000 kb to maintain SNP independence. (3) An F-test value greater than 10 was applied to exclude SNPs with insufficient explanatory power; (4) SNPs directly associated with the outcome variable (P2 < 5e-8) were removed; (5) effect directions and alleles were aligned to ensure that the minor allele frequency of SNPs was >0.01, and palindromes and incompatible SNPs were removed.

## Determination of Outcome Data

GWAS data for oral ulcers were obtained from the UK Biobank (Dataset ID: UKB-B-6458), which includes 47,102 cases and 414,011 control subjects (https://gwas.mrcieu.ac.uk/datasets/ukb-b-6458).

## Mendelian Randomization Analysis

In this study, MR analyses were performed using the Two-Sample MR software package to assess the potential causal relationship between genes and RAU. The inverse variance weighting (IVW) method was primarily used to assess the association between gene exposure and disease outcomes. To ensure the robustness and reliability of the results obtained through different methods, as well as to identify potential sources of bias, several sensitivity analyses were performed. These included MR-Egger regression, the simple model, the weighted median method, and the weighted model. This study followed a rigorous triple-criteria process to identify genes associated with RAU. First, statistically significant SNPs with p<0.05 in IVW analysis were initially screened. Then, these candidate instrumental variables were further refined by comparing the directional consistency of effect estimates across sensitivity analysis methods, i.e., odds ratios (ORs) were in the same direction (both >1 or both <1) in all methods. Finally, to exclude the effect of pleiotropy, SNPs that demonstrated statistical significance (p<0.05) in the pleiotropy test were excluded.

## Screening of Disease Critical Genes

The oral ulcer DEGs screened from the transcriptome data and the RAU-related genes from the MR analysis were cross-identified to obtain significant critical genes. These critical genes were visualized using a Venn Diagram. Subsequently, all critical genes were analyzed separately by MR to determine their causal relationship with the disease. The robustness and reliability of the findings were evaluated through heterogeneity analysis, multiple validity assessments, and leave-one-out sensitivity testing. Additionally, scatter plots, forest plots, and funnel plots were generated to visually present and substantiate the results.

## GO/KEGG Enrichment Analysis

Gene Ontology (GO) and Kyoto Encyclopedia of Genes and Genomes (KEGG) enrichment analyses were performed on critical differentially expressed genes using the clusterProfiler R package. The GO analysis encompassed three domains: biological processes (BP), cellular components (CC), and molecular functions (MF), while the KEGG analysis focused on identifying relevant signaling pathways involved in the pathogenesis of oral ulcers. Data visualization was carried out using the ggplot2 package, with statistical significance determined at an adjusted p<0.05.

## GSEA Enrichment Analysis 

Gene Set Enrichment Analysis (GSEA) was employed to investigate the biological relevance of critical genes. The analysis utilized the reference dataset “c2.cp.kegg.v7.2.symbols. gmt gene sets” from the MSigDB database. Gene sets meeting the criteria of a False Discovery Rate (FDR) < 0.25 and p<0.05 were regarded as statistically significantly enriched.

## Immune Cell Analysis

The infiltration levels of 22 immune cell types in RAU were analyzed using the CIBERSORT method. This approach aimed to uncover the relationship between critical RAU genes and immune cell infiltration, while also exploring the potential mechanisms by which these critical genes regulate immune cell behavior.

### RESULTS

#### Identification of DEGs

**Fig 1:**
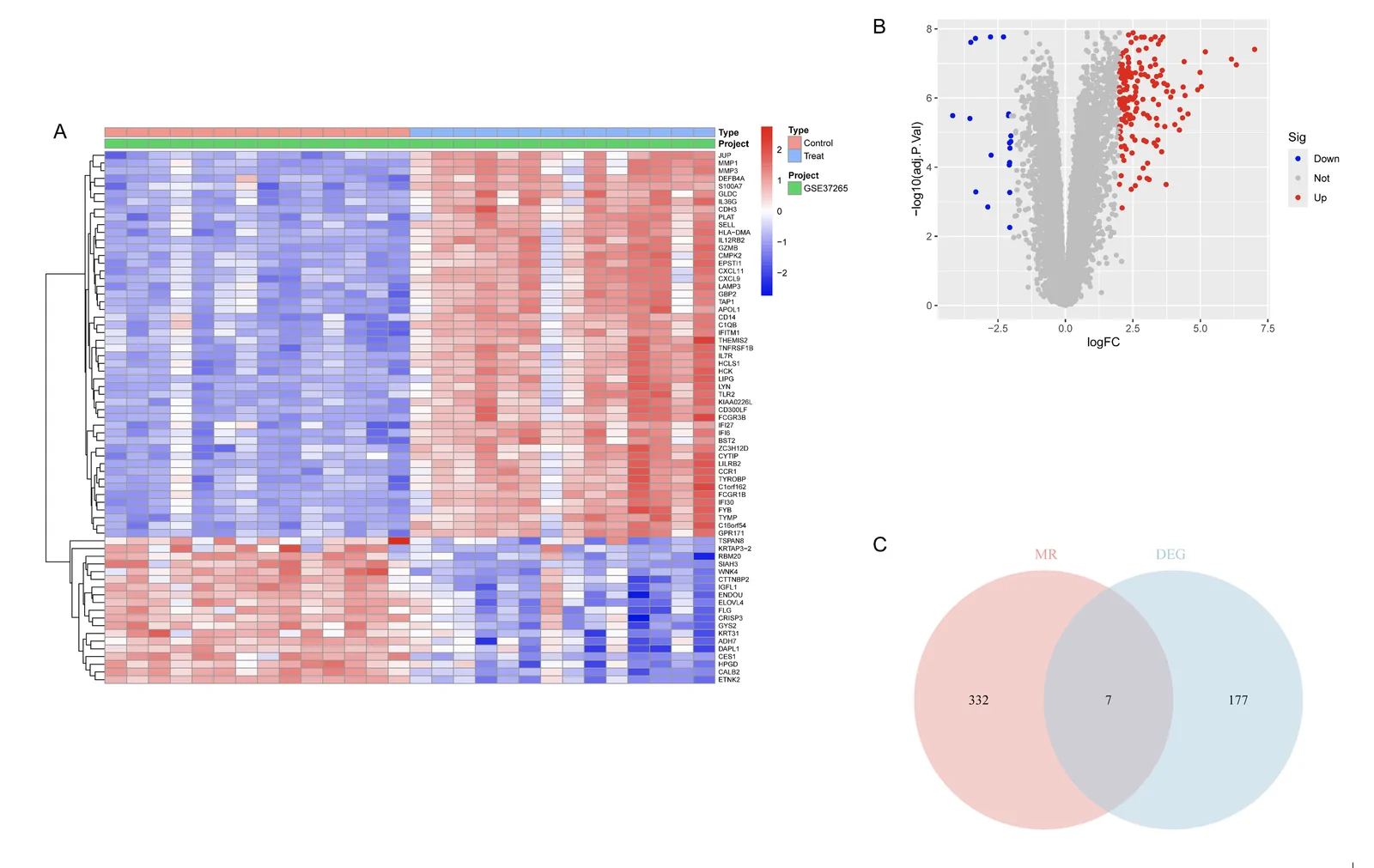
(A) Heatmap of differential gene expression (B) Differential gene volcano plot showing genes up-regulated in red and down-regulated in green (C) Veen plot showing the screening of 7 critical genes.

Ultimately, 184 DEGs were identified by transcriptomic data (Supplementary Table A1 provides detailed information (https://www.quintessence-publishing.com/quintessenz/journals/articles/downloads/ohpd_2026_8235_fu_appendix_table_1.xlsx). Heatmaps and volcano maps were used to visualize the differential genes (Figs 1A and 1B).

#### Mendelian Randomization Analysis

Through rigorous screening, 26,152 SNPs meeting the three core assumptions of MR were included as instrumental variables, and all had F-statistics > 10 (Table A2 ). Through MR analysis and application of the corresponding filtering criteria, 339 genes associated with RAU (Table A3 details the included data) were identified. Subsequently, the 184 differentially expressed genes (DEGs) obtained from the GEO database analysis were cross-referenced with the 339 genes obtained from the MR analysis. Visualization was performed by Venn diagrams (Fig 1C), identifying seven critical genes that were common to both sets of results: CCR1, CD177, ERP27,HCK, IFITM1, MICB, and SLC2A3.

**Fig 2:**
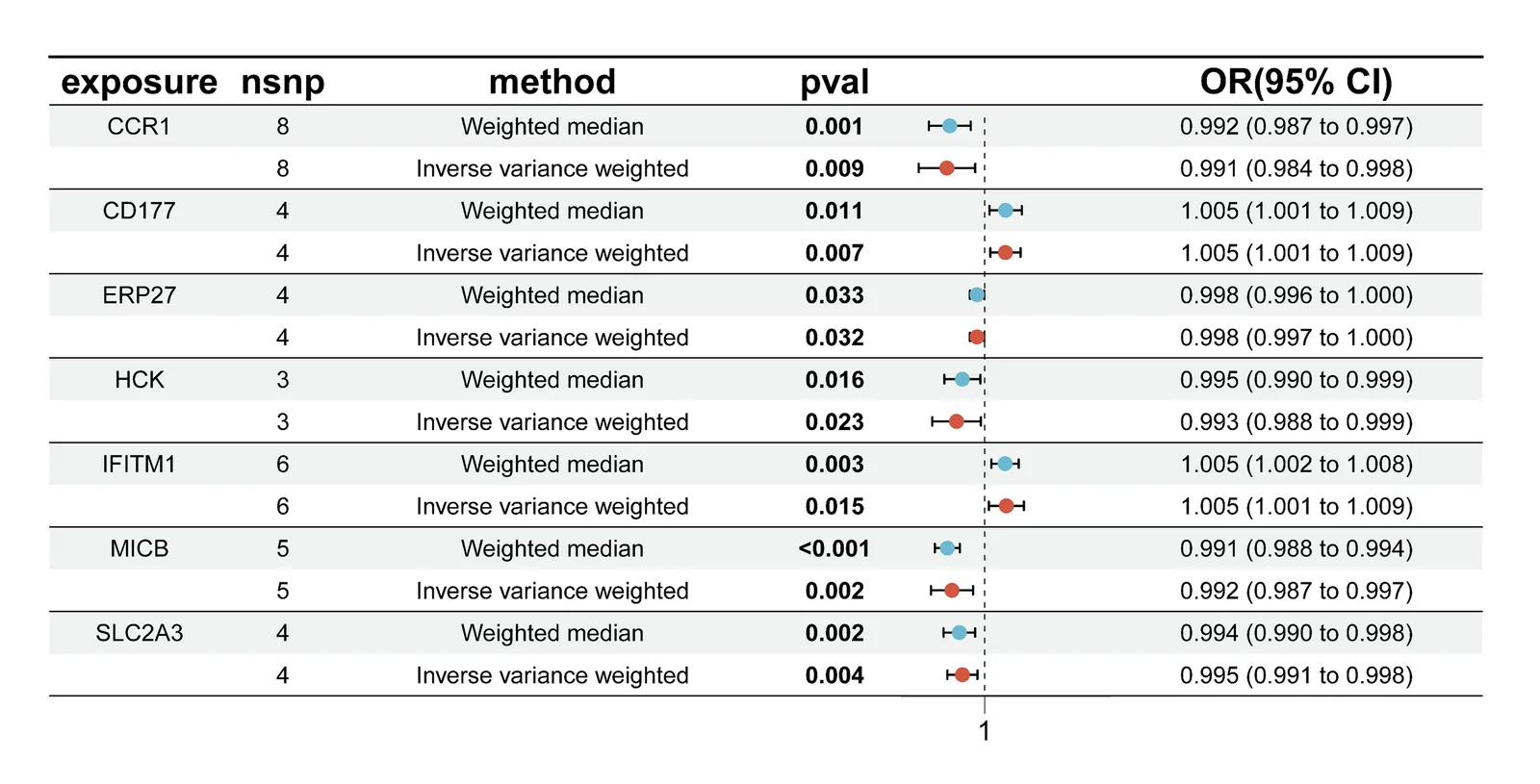
MR estimation of the relationship between 7 critical genes and RAU.

Next, MR analyses were performed on these seven key genes to assess their causal relationships with RAU. IVW analysis showed that CCR1 [OR = 0.991; 95% CI, 0.984–0.998; p=0.009], ERP27 [OR = 0.998; 95% CI, 0.997–1.000; p=0.032], HCK [OR = 0.993; 95% CI, 0.988–0.999; p=0.023], MICB [OR = 0.992; 95% CI, 0.987–0.997; p=0.002], and SLC2A3 [OR = 0.995; 95% CI, 0.991–0.998; p=0.004] were significantly associated with a decreased risk of RAU. In contrast, CD177 [OR = 1.005; 95% CI, 1.001–1.009; p=0.007] and IFITM1 [OR = 1.005; 95% CI, 1.001–1.009; p=0.015] were statistically significantly associated with an increased risk of RAU (Fig 2).

**Fig 3:**
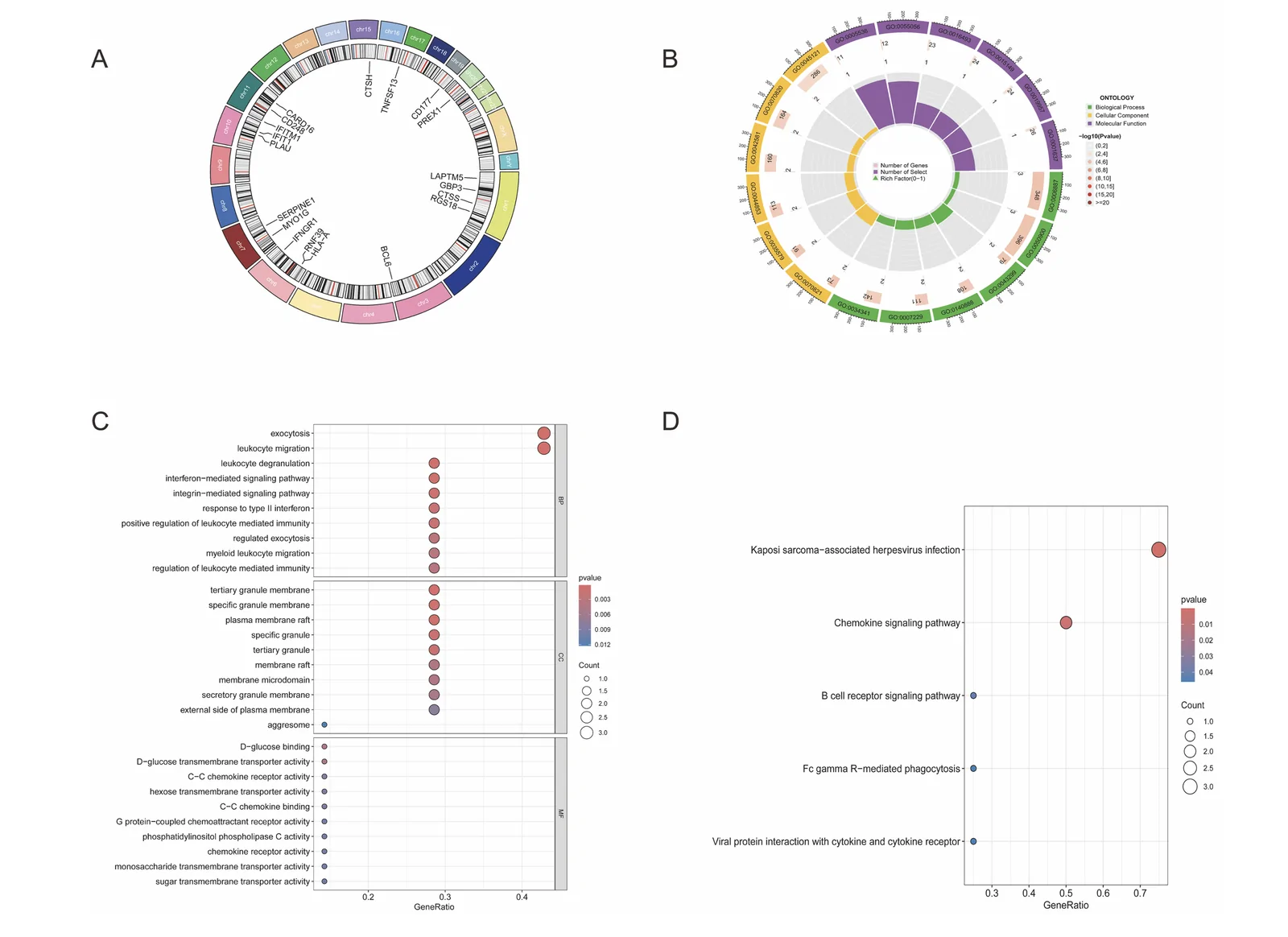
(A) Chromosome loops of the 7 critical genes; (B) GO circle ring plots of the 7 critical genes; (C) GO bubble plots of 7 critical genes; (D) KEGG bubble plots of 7 critical genes.

The critical differential genes were subsequently visualized using a chromosome circular map (Fig 3A), and the results demonstrated the specific locations of these seven genes on the chromosome: ERP27 and SLC2A3 are located on chromosome 12, CD177 is located on chromosome 19, HCK is located on chromosome 20, CCR1 is located on chromosome 3, MICB is located on chromosome 6, and IFITM1 is located on chromosome 11 .

The sensitivity analysis results (Table 1) indicated the absence of heterogeneity or horizontal pleiotropy for these critical genes (p>0.05). Furthermore, the symmetry observed in the funnel plot suggested that no confounding factors influenced the causal relationship between eQTL expression levels and RAU. Leave-one-out sensitivity analysis confirmed that excluding any single SNP did not lead to statistically significant changes in the MR analysis outcomes, and the effect sizes of individual instrumental variables were consistent with the overall effect sizes. These findings collectively underscore the robustness of the analyses. Comprehensive details for each gene, including funnel plots, scatter plots, and leave-one-out sensitivity analyses, are provided in Supplementary Figs A1-A3. (https://www.quintessence-publishing.com/quintessenz/journals/articles/downloads/ohpd_2026_8235_fu_appendix_table_1.xlsx)

**Table 1:** Details of the sensitivity analysis of the seven critical genes to the MR results of RAU

Exposures	Heterogeneity test						Pleiotropy test		
	MR-Egger			Inverse variance-weighted			MR-Egger		
	Q	Q_df	Q_P	Q	Q_df	Q_P	Intercept	SE	P
CCR1	54.1595	6	6.8500E-10	54.3438	7	2.0100E-09	-0.0002	0.0017	0.891
CD177	2.9023	2	0.2343	2.9707	3	0.3962	0.0003	0.0012	0.8482
ERP27	1.8414	2	0.3982	1.8867	3	0.5962	-0.0002	0.001	0.8512
HCK	2.7975	1	0.0944	4.4854	2	0.1062	-0.001	0.0013	0.5796
IFITM1	8.9307	4	0.0629	9.3203	5	0.0969	0.0004	0.0001	0.6976
MICB	12.472	3	0.0059	15.0277	4	0.0046	0.0022	0.0028	0.4902
SLC2A3	2.2838	2	0.3192	3.2165	3	0.3594	0.0006	0.0007	0.4615


#### GO and KEGG Enrichment Analysis

GO and KEGG analyses were conducted to further investigate the potential functions of these seven critical genes. GO analyses revealed that at the biological process (BP) level, these genes were enriched in multiple processes associated with immune response and cell migration. Cellular component (CC) analysis revealed multiple functional regions associated with secretory granules and membrane structure. In terms of molecular function (MF), multiple genes were involved in the binding and transport of sugars and their derivatives (Figs 3B and 3C) KEGG pathway enrichment analysis revealed that these critical genes were statistically significantly enriched in multiple key pathways associated with immunity and viral infection (Fig 3D). Detailed data for GO and KEGG can be found in Tables A4 and A5.

#### GSEA Enrichment Analysis

**Fig 4:**
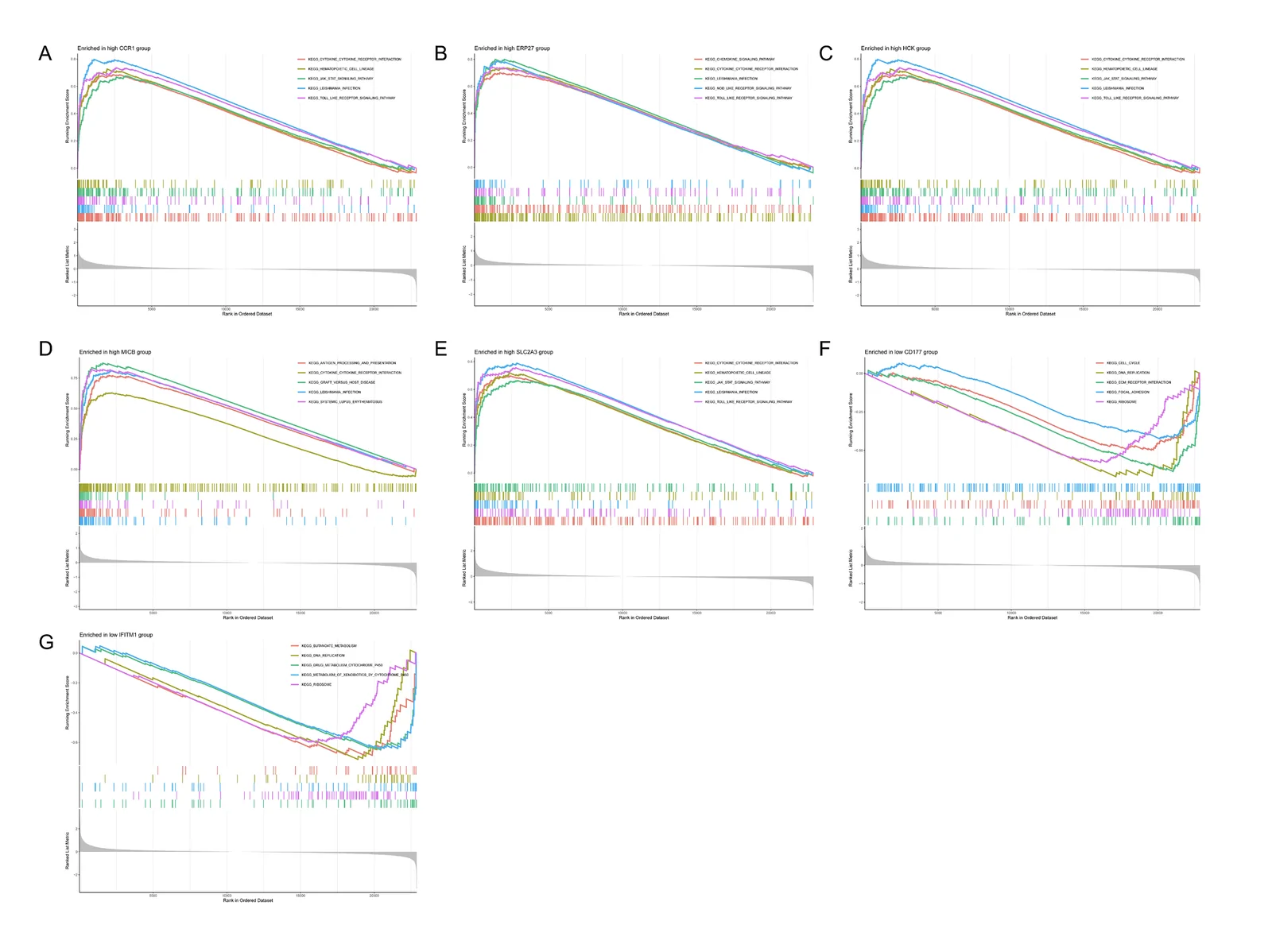
(A-E) GSEA enrichment results for high expression of CCR1, ERP27, HCK, MICB, and SLC2A3 (F-G) GSEA enrichment results for low expression of CD177 and IFITM1.

GSEA revealed that the low-risk group, characterized by high expression of CCR1, ERP27, HCK, MICB, and SLC2A3 and low expression of CD177 and IFITM1, was mainly enriched in immune- and inflammation-related pathways, such as cytokine–cytokine receptor interaction and the Toll-like receptor signaling pathway (Figs 4A to 4G).

**Fig 5:**
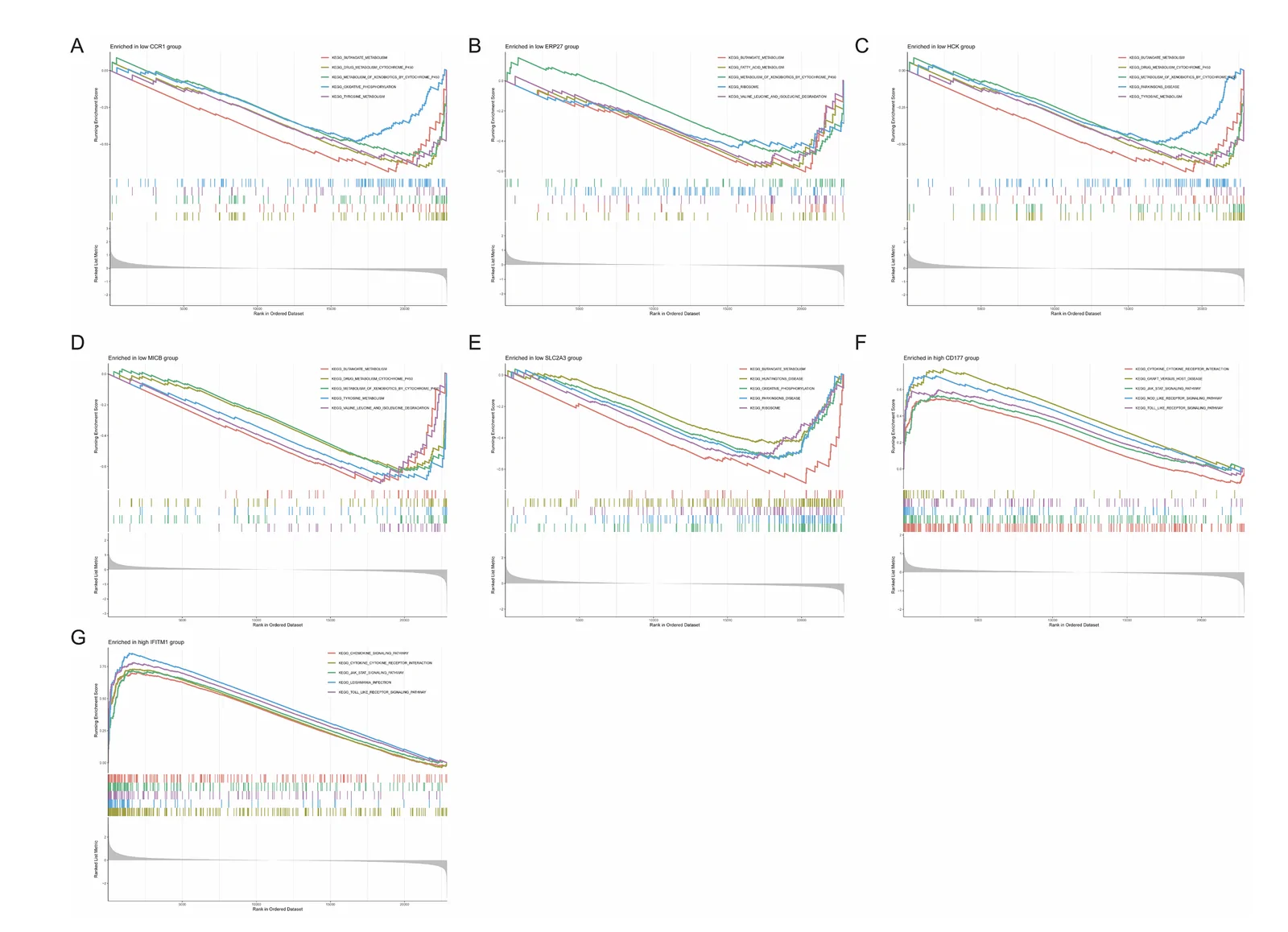
(A-E) GSEA enrichment results for low expression of CCR1, ERP27, HCK, MICB, and SLC2A3 (F-G) GSEA enrichment results for high expression of CD177 and IFITM1.

In the high-risk group (high expression of CD177 and IFITM1, low expression of CCR1, ERP27, HCK, MICB, SLC2A3), metabolism-related pathways were statistically significantly activated, including for instance butanoate metabolism, tyrosine metabolism, oxidative phosphorylation, etc (Figs 5A to 5G).

#### Assessment of RAU Immune Cell Infiltration

**Fig 6:**
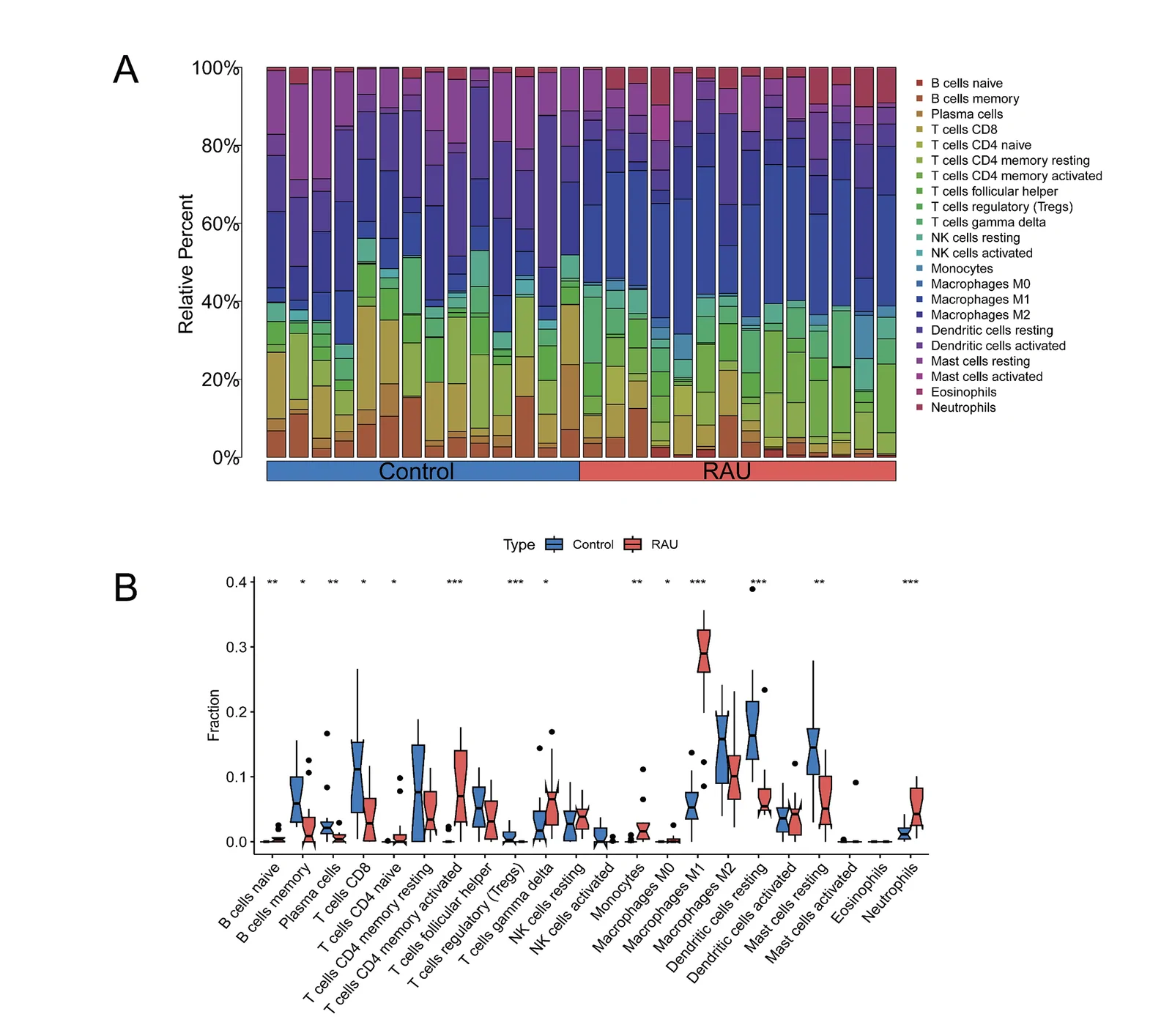
Immune cell infiltration analysis in RAU. (A) Stacked histogram illustrating the proportions of immune cells in the RAU and control groups; (B) Box-plot comparing immune cell distributions between the two groups; (C) Correlation analysis between seven critical genes and immune cell levels (*p<0.05, **p<0.01, ***p<0.001).

Enrichment analysis of critical genes in RAU showed a close correlation with the immune process. We then analyzed the relative percentages of different types of immune cells between RAU disease states and controls by CIBERSORT, which showed statistically significant differences in immune cell subpopulations between the two groups, especially B-cells, T cells, natural killer cells and macrophages (Figs 6 A and 6B). This suggests that these immune cell types play a statistically significant role in the development of RAU.

Correlation analysis further revealed statistically significant associations between the seven key genes and immune cell subsets (Fig 6C). Except for CD177, all key genes showed strong correlations with multiple immune cell types. Specifically, HCK, CCR1, IFITM1, and MICB were negatively associated with memory B-cells and follicular helper T-cells, whereas HCK, IFITM1, SLC2A3, and MICB were positively associated with activated CD4 memory T-cells. In addition, strong correlations were observed among several immune cell subsets, including memory B-cells vs naive B-cells and resting mast cells vs activated dendritic cells, implying potential interactions among these immune populations.

### DISCUSSION

In this work, the critical disease-causing genes of RAU were systematically explored for the first time by using a combination of transcriptomics and MR analysis, which provided a new perspective for understanding its pathogenesis.

Through data integration and analysis, we identified a total of 184 DEGs, which were cross-referenced with 339 RAU-associated genes identified through MR analysis, and finally screened seven statistically significant critical genes. Among these genes, CCR1, ERP27, HCK, MICB, and SLC2A3 were negatively associated with RAU risk, whereas CD177 and IFITM1 were statistically significantly associated with increased RAU risk. This finding provides new clues to the pathomechanism of RAU and lays the foundation for potential targeted therapeutic strategies.

#### Biological Significance of Critical Genes

Functional analysis of the critical genes showed that CCR1, as a chemokine receptor, was mainly expressed in a variety of immune cells, and may regulate immune responses by directing immune cell migration.^14,21,31^ Its negative correlation with RAU risk suggests that CCR1 may play a protective role in controlling inflammation and maintaining immune homeostasis; ERP27, as an endoplasmic reticulum stress-associated protein, may attenuate cellular damage and inflammatory responses by promoting the degradation of abnormally folded proteins.^1,8,37^

HCK serves a critical function in the regulation of immune signaling and cell motility, and its negative correlation with the risk of RAU suggests that HCK may help maintain the balance of the local immune response by suppressing excessive inflammation.^6,15,38^ MICB, as a major histocompatibility complex-related gene that activates natural killer cells, suggesting its role in enhancing local immune responses.^9,24,27^ In contrast, CD177 and IFITM1 may exacerbate the inflammatory response in RAU by promoting the recruitment and activation of immune cells.^7,19^

#### Immune-metabolic Interactions

GO enrichment analysis indicated that critical genes are related to a variety of biological processes related to immune regulation and cell migration, including exocytosis, leukocyte migration, and leukocyte degranulation. These biological processes are closely related to the activation and migration of immune cells and the release of inflammatory factors, indicating that critical genes have important functions in immune regulation. In addition, enrichment of the interferon-mediated signaling pathway suggests that these genes may also participate in interferon signaling triggered by viral infection or inflammation.

From the perspective of cellular composition, these critical genes were mainly enriched in the “tertiary granule membrane”, “specific granule membrane “specific granule membrane” and “membrane raft”, reflecting their possible involvement in substance transport and signaling within immune cells.^5,16,25,32^ Meanwhile, molecular functional analysis revealed that critical genes were closely related to sugar binding and transmembrane transporter, such as “D-glucose binding” and “sugar transmembrane transporter activity”. This functional characterization suggests that the related genes are important in maintaining cellular metabolic homeostasis and energy homeostasis.^12,28,35^ In addition, the important role of glucose metabolism in the activation, proliferation, and functional regulation of immune cells has been extensively investigated, which further supports the critical role of these genes in immune response, especially in diseases involving metabolism-immunity interactions with potential impact.^20,36^

KEGG pathway analysis further revealed that the critical genes were involved in multiple signaling pathways closely related to immune response and pathogen infection. These include Kaposi sarcoma-associated herpesvirus infection, chemokine signaling pathway, B cell receptor signaling pathway cell receptor signaling pathway) and Fc gamma R-mediated phagocytosis. The activity of these signaling pathways suggests that the function of genes may provide host defense against inflammation and infection by regulating immune response mechanisms.^2,3,11,30^

GSEA further revealed functional differences between the high- and low-risk groups. Specifically, GSEA demonstrated distinct functional characteristics of RAU critical genes in the two groups. In the high-risk group, relevant genes were statistically significantly enriched in immune and inflammation-related signaling pathways. Among these pathways, the JAK-STAT signaling pathway plays an important role in cell proliferation, differentiation, and immune regulation. Its aberrant activation is thought to exacerbate pathological inflammatory responses.^34^ The chemokine signaling pathway is closely related to the recruitment of immune cells and regulation of the inflammatory microenvironment.^22^ Overactivation of these immune pathways may lead to excessive inflammatory responses in the oral mucosa of patients with RAU. This may impair tissue repair and promote recurrent ulceration. In contrast, genes in the low-risk group were statistically significantly enriched in metabolic regulation-related pathways. Fatty acid metabolism and butyric acid metabolism have key roles in maintaining cellular energy supply and mucosal barrier integrity.^23^ In addition, downregulation of oxidative phosphorylation pathways may reflect reduced metabolic activity. This change may help decrease the metabolic burden and thereby prevent excessive inflammatory activation.^13^ The function of these metabolism-related pathways may balance immune effects by maintaining metabolic homeostasis, thereby reducing the risk of RAU recurrence. Notably, cell cycle- and DNA replication-related pathways were also significantly enriched in the low-risk group. This finding may indicate that these pathways promote cell proliferation and tissue repair.^39^

#### Changes in the Immune Microenvironment

CIBERSORT analysis revealed statistically significant differences in the distribution of immune cell subpopulations between RAU patients and controls. In particular, B-cells, T-cells, natural killer cells, and macrophages showed marked differences in abundance. These findings suggest that these immune cells may act as pivotal factors in the development and progression of RAU. Correlation analysis revealed that seven critical genes were statistically significantly associated with multiple immune cell types. For example, HCK and CCR1 were negatively correlated with memory B-cells and follicular helper T-cells. In contrast, HCK and IFITM1 were positively correlated with activated CD4 memory T-cells. These findings suggest that these genes may influence the immunopathological features of RAU by regulating the activation and function of specific immune cell subsets.

In addition, the study demonstrated synergistic interactions among immune cells, such as positive correlations between memory B-cells and naïve B-cells, and between resting mast cells and activated dendritic cells, reflecting the complexity and highly coordinated nature of the immune system. These findings provide important clues for further exploration of the changing patterns of the immune microenvironment of RAU and its mechanism of action in the disease.

#### Study Limitations

Despite the strengths of integrating transcriptomic analysis with MR-based gene prioritization, several limitations should be acknowledged. First, the GSE37265 dataset included a relatively small sample size (14 RAU samples and 14 controls), which may have limited the statistical power of the differential expression analysis and increased the risk of false-negative or unstable findings. Although we applied relatively stringent thresholds to improve the robustness of DEG identification, some potentially relevant genes with moderate expression changes may have been missed. Second, this study was primarily based on public datasets and bioinformatics inference, and the identified associations do not by themselves establish direct biological causality. Third, immune infiltration estimation and pathway enrichment analyses were computationally derived and may be influenced by dataset heterogeneity and algorithmic assumptions. Therefore, the present findings should be interpreted with caution and require further validation in larger, independent cohorts as well as experimental studies.

#### Clinical Implications and Translational Prospects

Although the present study is exploratory, the identified critical genes may have potential translational relevance in RAU. On the one hand, genes such as CCR1, HCK, IFITM1, CD177, and SLC2A3 may serve as candidate biomarkers for disease risk stratification, inflammatory activity assessment, or recurrence monitoring. On the other hand, the enrichment of these genes in immune- and metabolism-related pathways suggests that they may represent potential therapeutic targets for modulating the inflammatory microenvironment and promoting mucosal healing. In particular, pathways such as JAK–STAT signaling, chemokine signaling, and Fcγ receptor-mediated immune responses may provide mechanistic entry points for targeted intervention. However, before clinical application, these findings require validation in larger multicenter cohorts and in clinically accessible samples. Further functional experiments are also needed to confirm the biological roles of these genes and to determine whether a multi-gene panel could improve diagnostic accuracy and therapeutic decision-making in RAU.

Overall, the multilevel analysis of this study reveals the complex interplay between immune response and metabolic regulation in RAU disease mechanisms. The central role of immune regulation is reflected in the aberrant activation of immune cell activity and signaling pathways, whereas dysregulation of metabolism-related functions may affect the energy requirements of immune cells and the repair efficiency of damaged tissues. The combined effects of the two may collectively accelerate the inflammatory response and exacerbate the course of the disease. These findings not only provide new insights into the pathomechanism of RAU, but also suggest potential directions for intervention. Given that both immune function and metabolic regulation have important roles in RAU, future therapeutic strategies should focus on modulating the aberrant activity of the immune system while balancing metabolic processes to promote tissue repair. In addition, further experimental validation targeting key pathways (e.g., JAK-STAT signaling, chemokine signaling, and Fcγ receptor-mediated function) and critical genes (e.g., HCK, IFITM1, CCR1, etc) will contribute to better designs of precise therapeutic regimens targeting patients with RAU.

### CONCLUSIONS

Throughout this study, critical genes associated with RAU were identified and their potential biological mechanisms by a combination of transcriptome data analysis and Mendelian randomization. A total of seven significant critical genes were identified, of which CCR1, ERP27, HCK, MICB and SLC2A3 were negatively associated with RAU risk, whereas CD177 and IFITM1 were statistically significantly associated with increased RAU risk. These findings reveal the complexity of the interaction between immune response and metabolic regulation in RAU pathology, suggesting potential targets for intervention and therapeutic ideas. Future studies should continue to explore the functions of the genes and their roles in the pathogenesis of RAU, with the aim of providing new targeting strategies for clinical treatment and thus improving the quality of life of patients.

### ACKNOWLEDGMENTS

This work was supported by the National Natural Science Foundation of China (NSFC) (82060202) and Youth Science Foundation of Guangxi Medical University (GXMUYSF202545), and the Natural Science Foundation of Guangxi Zhuang Autonomous Region (No. 2025GXNSFAA069622). We would like to express our sincere gratitude to all the doctors from the Department of Preventive Dentistry at the Affiliated Stomatological Hospital of Guangxi Medical University for their invaluable support and assistance throughout this research. Their expertise greatly contributed to the successful completion of this study. We also appreciate the feedback from our peers, which enhanced the quality of our work.

#### AI Statement

We utilized AI tools for language refinement and proofreading throughout the manuscript. Specifically, AI was employed to enhance clarity, ensure grammatical accuracy, and improve overall readability. All content was reviewed to meet the publication standards of Quintessence Publishing, ensuring adherence to academic integrity.

### REFERENCES
